# DR30318, a novel tri-specific T cell engager for Claudin 18.2 positive cancers immunotherapy

**DOI:** 10.1007/s00262-024-03673-x

**Published:** 2024-03-30

**Authors:** Zhe Ma, Zhenxing Zhou, Wenwen Duan, Gaofeng Yao, Shimei Sheng, Sidou Zong, Xin Zhang, Changkui Li, Yuanyuan Liu, Fengting Ou, Maha Raja Dahar, Yanshan Huang, Lushan Yu

**Affiliations:** 1 Institute of Drug Metabolism and Pharmaceutical Analysis, College of Pharmaceutical Sciences, Zhejiang University, Hangzhou, 310058 Zhejiang Province China; 2 Department of Innovative Drug Discovery and Development, Zhejiang Doer Biologics Co., Ltd., Hangzhou, 310058 Zhejiang Province China; 3 National Key Laboratory of Advanced Drug Delivery and Release Systems, Zhejiang University, Hangzhou, 310058 China; 4 Department of Pharmacy, Second Affiliated Hospital, School of Medicine, Zhejiang University, Hangzhou, 310009 China; 5 Jinhua Institute of Zhejiang University, Jinhua, 321036 China; 6 Department of Pharmacy, Shaoxing People’s Hospital (Shaoxing Hospital, Zhejiang University School of Medicine), Shaoxing, 312000 China

**Keywords:** Claudin 18.2, T cell engager, Gastric cancer, Pancreatic cancer, Cytokines, TDCC

## Abstract

**Background:**

Claudin 18.2 (CLDN18.2) is a highly anticipated target for solid tumor therapy, especially in advanced gastric carcinoma and pancreatic carcinoma. The T cell engager targeting CLDN18.2 represents a compelling strategy for enhancing anti-cancer efficacy.

**Methods:**

Based on the in-house screened anti-CLDN18.2 VHH, we have developed a novel tri-specific T cell engager targeting CLDN18.2 for gastric and pancreatic cancer immunotherapy. This tri-specific antibody was designed with binding to CLDN18.2, human serum albumin (HSA) and CD3 on T cells.

**Results:**

The DR30318 demonstrated binding affinity to CLDN18.2, HSA and CD3, and exhibited T cell-dependent cellular cytotoxicity (TDCC) activity in vitro. Pharmacokinetic analysis revealed a half-life of 22.2–28.6 h in rodents and 41.8 h in cynomolgus monkeys, respectively. The administration of DR30318 resulted in a slight increase in the levels of IL-6 and C-reactive protein (CRP) in cynomolgus monkeys. Furthermore, after incubation with human PBMCs and CLDN18.2 expressing cells, DR30318 induced TDCC activity and the production of interleukin-6 (IL-6) and interferon-gamma (IFN-γ). Notably, DR30318 demonstrated significant tumor suppression effects on gastric cancer xenograft models NUGC4/hCLDN18.2 and pancreatic cancer xenograft model BxPC3/hCLDN18.2 without affecting the body weight of mice.

**Supplementary Information:**

The online version contains supplementary material available at 10.1007/s00262-024-03673-x.

## Introduction

Gastric cancer, ranked as the sixth most prevalent cancer globally, is the third leading cause of cancer-related mortality [1], and is widely recognized as one of the most challenging malignancies to treat. Although early gastric cancer exhibits a 5-year overall survival rate (OSR) exceeding 90% in Asia [2–5], the OSR for advanced gastric cancer remains low due to late-stage diagnosis in over 80% of patients [6]. Pancreatic cancer is characterized by a dismal prognosis and high mortality, making it the most lethal disease. In 2019, the survival rate of patients diagnosed with pancreatic adenocarcinoma was 24% at 1 year and only 9% at 5 years in the USA and Europe in 2019 [1, 7]. The relatively low survival rate can be partly attributed to the prevalence of advanced-stage diagnosis in most cases, as only approximately 20% of patients are diagnosed at an early stage when surgical resection is feasible [8]. Furthermore, even after surgery without additional therapy, more than 90% of patients experience disease relapse and succumb to their illness [9].

Currently, the market for gastric cancer immunotherapy is limited to HER2-targeted drugs [10–12], anti-PD-(L)1 agents [13, 14] and anti-VEGFR treatments [15]. However, these therapies have shown only modest benefits in gastric/GEJ cancer patients, with objective response rates (ORR) ranging between 3 and 11% [14, 16]. For pancreatic cancer, although monoclonal antibodies targeting integrin α3, Mucins, EGFR, TROP2 and α6β4 have demonstrated efficacy in pancreatic tumor xenograft models, their clinical translation has been limited with minimal survival benefits observed in patients [17–19]. Therefore, to address the unmet need for effective antibody-based therapies in gastric and pancreatic cancer treatment, the identification of a novel promising target is imperative.

Claudin 18 (CLDN18) is a member of the Claudin family with four transmembrane domains and has two splice variants, CLDN18.1 and CLDN18.2. In normal tissues, CLDN18.1 is strictly expressed on epithelial cells of lung tissue, while CLDN18.2 is exclusively expressed on differentiated gastric mucosal cells [20]. Notably, CLDN18.2 exhibits significantly aberrant expression in multiple cancers, such as gastric cancer [21–23], pancreatic cancer [24] and esophageal adenocarcinomas [25]. Given its specific expression pattern, targeting CLDN18.2 holds great promise for therapeutic interventions against gastric and pancreatic cancer. Zolbetuximab (IMAB362), which exerts potent antitumor activity through antibody-dependent cytotoxicity (ADCC) and complement-dependent cytotoxicity (CDC), is the world's first monoclonal antibody drug targeting CLDN18.2 [26]. In 2023, Astellas reported the exceptional efficacy of Zolbetuximab in phase III clinical trials for advanced unresectable or metastatic gastric or gastroesophageal junction (mG/GEJ) adenocarcinoma patients, particularly among Asian populations [27–29]. Meanwhile, various therapeutic modalities targeting CLDN18.2 are currently under investigation, including monoclonal antibody [30], bispecific antibody [31], CAR-T cell therapy and other approaches [32].

The bispecific T cell engager (BiTE) specifically targets the tumor cell antigen and CD3 on T cells, thereby inducing the potent T cell-dependent cellular cytotoxicity (TDCC). However, in terms of canonical BiTE, continuous intravenous infusion is needed to maintain therapeutic concentration due to its rapid clearance [33]. The antibody isotype immunoglobulin G (IgG) and human serum albumin (HSA) have longer plasma half-life of about 3 weeks in human body [34]. Strategies, based on IgG-Fc backbone or affinity reagents that recognize and bind HSA, can be used to extend the half-life of BiTE [35–37]. AMG910 is the first bispecific T cell engager targeting CLDN18.2 based on Amgen’s half-extended (HLE) BiTE platform. A phase I clinical trial investigating AMG910 for patients with CLDN18.2 positive cancers is underway [38]. In this study, we developed a tri-specific T cell engager DR30318 based on the MultipleBody® platform of Doer Biologics, specifically targeting CLDN18.2, HSA and CD3. The in vitro and in vivo studies demonstrated that DR30318 exhibited potent TDCC activity and robust efficacy in suppressing tumor growth. Pharmacokinetic (PK) studies revealed a linear PK profile in cynomolgus monkeys. The lower degree of cytokine release and robust tumor regression activity position DR30318 as a promising therapeutic agent for CLDN18.2 positive cancers.

## Materials and methods

### Cell lines

CHO-K1Q cells were cultured in CD02 medium (Quacell), BxPC3 and HEK293 cells were cultured in high glucose DMEM medium (BasalMedia) supplied with 10% fetal bovine serum (FBS, ExcellBio), Panc1, NUGC4 and SNU620 cells were cultured in RPMI 1640 medium (BasalMedia) supplied with 10% FBS.

For construction of CLDN18.2 over-expressing cells, the cDNA under the control of CMV promoter was cloned into the plasmid pDR05 or pBX to obtain the plasmids pDR05-CLDN18.2 and pBX-CLDN18.2, respectively. CHO-C18.2-gfpx cells were obtained by electroporation with pDR05-CLDN18.2, and under the selection of glutamine synthetase (GS)-based system. Panc1-C18.2, NUGC4-C18.2 and SNU620-C18.2 cells were obtained by transfection with Lipo3000 (Thermofisher) and selected under the pressure of puromycin (Invivogen).

### Generation of DR30318

DR30318 is consisting of an in-house screened anti-CLDN18.2 variable domains of heavy chain of heavy-chain antibodies (VHH) from an *alpaca*, an anti-HSA nanobody and an anti-CD3 single-chain fragment variable (scFv). The gene sequence encoding DR30318 was cloned into the pDR03 vector to obtain the expression plasmid. Subsequently, transient transfection of HEK293 cells with pDR03-30318 was performed using PEI as a transfection reagent. The cultural supernatant containing DR30318 protein was collected and subjected to purification through affinity chromatography (Bestchrom Biosciences, AT Protein A Diamond). The purified protein underwent preliminary verification by SDS-PAGE (about 52 kDa) and Size Exclusion Chromatography 300 A (Agilent). Meanwhile, the benchmark AMG910 analog was expressed based on the sequence information released by Amgen (US11692031B2).

### In vitro binding assay

The binding affinity of DR30318 to CLDN18.2 or CD3 was assessed using flow cytometry (FCM) method. CHO-CLDN18.2-gfpx cells (exogenously over-expressing the CLDN18.2 protein) or Jurkat cells were harvested and adjusted to 5 million cells/ml with assay buffer (RPMI 1640 supplemented with 1% FBS). The cell suspension was seeded into a round-bottomed 96-well plate, and serially diluted DR30318 was added and incubated for one hour at 4 °C. After two washes with PBS, the cells were further incubated with rabbit anti-VHH polyclonal antibody and fluorescent secondary antibody before being rewashed with PBS. Finally, the cell suspension was analyzed using a NovoCyte flow cytometer (Agilent, 2060R).

The binding affinity of DR30318 to serum albumin of human (HSA) and bovine (BSA) was determined using an enzyme-linked immunosorbent assay (ELISA). Briefly, HSA or BSA was immobilized onto 96-well microplates for the capture of DR30318. Subsequently, anti-VHH rabbit polyclonal antibody and HRP-labeled secondary antibody were sequentially incubated. Following five washes with PBST, TMB substrate was added and incubated, and the optical density at 450 nm (OD_450_) was measured using a microplate reader.

### TDCC reporter assay

An in vitro TDCC reporter assay was conducted to evaluate the antitumor activity of DR30318. Jurkat-NFAT-luc2p-10F2 (effector cells) and CLDN18.2 expression cells (target cells) such as CHO-C18.2-gfpx, BxPC3, Panc1, Panc1-C18.2, SNU620, SNU620-C18.2 and NUGC4-C18.2 were harvested, adjusted to 2.4 × 10^6^/ml and 6 × 10^5^/ml, respectively, and mixed at a ratio of 1:1 by volume. The mixture was seeded into a 96-well plate with 50 μl/well, followed by the addition of serially diluted DR30318 or other reference proteins at a volume of 50 μl/well. After incubation for 18 h at 37 °C, cell cultures were measured using the Bright-Glo™ Luciferase Assay System (Promega, E2620), according to the manufacturer's instructions.

### PBMC-based TDCC assay

The antitumor activity of DR30318 was also assessed in vitro using the PBMC-based TDCC assay. T cells isolated using the Invitrogen™ MagniSort™ Human T cell Enrichment Kit (Invitrogen, 8804-6810-74) or the peripheral blood mononuclear cells (PBMCs) of two healthy donors and target cells BxPC3, Panc1, Panc1-C18.2, SNU620, SNU620-C18.2 and NUGC4-C18.2 were harvested and adjusted to 4 × 10^6^/ml and 1 × 10^5^/ml, respectively, and mixed at a ratio of 1:1 by volume. The mixture was seeded into a 96-well plate with 50 μl/well, followed by the addition of serially diluted DR30318 or other reference proteins at a volume of 50 μl/well. The cell culture plates were further incubated for 24 h at 37 °C. Then, the lactate dehydrogenase (LDH) levels in the supernatant were measured according to the instructions provided with the Cytotoxicity LDH Assay Kit (Donjido, CK12).

### T cell stimulation assay

PBMCs of two healthy donors were seeded into 96-well U type plate and incubate with DR30318 or AMG910 analog for 0,3 or 7 days. Then, Alexa Fluor® 488 anti-Human CD3 (Biolegend, 317,310), PerCP anti-human CD8 (Biolegend, 344,708) and APC anti-human CD4 (Biolegend, 300,514) were applied for measurement of the BiTE—induced T cell proliferation.

### Pharmacokinetics

The pharmacokinetic (PK) profiles of DR30318 were evaluated in rodents and non-human primates.

C57BL/6 and NOD-SCID mice (four females and four males), aged 6–7 weeks, received a tail intravenous infusion of DR30318 at doses of either 0.1 or 1 mg/kg. Blood samples were collected before and after administration at 1, 6, 24, 30, 48, 72, 96 and 120 h.

Cynomolgus monkeys (one female and one male per group) were intravenously infused with DR30318 at 0.03, 0.6 or 3 mg/kg. Blood samples were collected before administration, immediately after administration, at 4, 8, 24, 48, 72, 120 and 168 h. For repeat-dosage of DR30318, cynomolgus monkeys (two females and two males) were given 0.03 mg/kg DR30318 once a week. Blood samples were collected before and immediately after administration, as well as at 4, 8, 24-, 48-, 72- and 120-h post-dosage for both the first and fourth administrations.

The serum concentration of DR30138 was determined with the ELISA method.

### Cytokines release

The serum samples from the 0.03 mg/kg and 3 mg/kg groups were analyzed for IL-2, IL-6, TNF-α and CRP.

To assess the potential risk of cytokines release syndrome (CRS) induced by DR30318 administration in the human study, the TDCC-based in vitro cytokine release assay was performed. In brief, CHO-C18.2-gfpx cells were used as target cells, and the PBMCs from two healthy donors were used as effector cells. 4 × 10^4^ cells of the target were incubated with 2 × 10^5^ cells of the effector and serially gradient concentration of DR30318. After being incubated at 37 °C and 5% CO_2_ for 24 h and 48 h, the culture plates were centrifugated at 1500 RPM for 5 min. The production of IL-2, IL-6, IL-10, TNF-α and IFN-γ in the supernatants was detected with LEGENDplex™ HU Th1 Panel (Biolegend, 741036).

### Xenograft tumor models

To establish the human gastric cancer or pancreatic cancer model, male NCG mice (GemPharmatech), aged 6–8 weeks, were subcutaneously injected with 5 × 10^6^ cells of gastric cancer cell line NUGC4/hCLDN18.2 or pancreatic cancer cell line BxPC3/hCLDN18.2. After 7 days, 2 × 10^6^ PBMC cells were intravenously infused via tail injection. Upon reaching an average tumor volume of approximately 40–60 mm^3^, the mice were randomly allocated into five groups, each group containing six mice, for intraperitoneal administration of DR30318: a vehicle control group receiving normal saline, BIW (twice a week) at doses of 0.03 mg/kg and 0.1 mg/kg, respectively, and QD (daily) at doses of 0.03 mg/kg and 0.1 mg/kg, respectively.

Tumor volume was measured twice weekly with a Vernier caliper and determined according to the following equation: Tumor volume (mm^3^) = 1/2 length × width^2^. After the mice were sacrificed, the tumors were dissected and photographed.

### Immunohistochemical staining

Tumor tissues from Xenograft tumor models were collected, fixed, paraffin-embedded and sectioned. The sections were floated onto clean glass slides, deparaffinized and antigen retrieval with microwave. The endogenous peroxidase activity was then blocked at room temperature by a 5–10 min incubation in the final developmental 3% H_2_O_2_ before being blocked with normal goat serum. The slides were then incubated with anti-Claudin 18 [34H14L15] antibody (abcam, ab203563) or anti-CD3 epsilon antibody (abcam, ab5690). Finally, the slides were counterstained with hematoxylin and recorded by Nikon microscope.

### Statistical analysis

All statistical analyses were conducted using the GraphPad Prism software. Experimental data were presented as mean ± standard deviation (SD) or mean ± standard error of the mean (SEM). Two-group comparisons were assessed using the student’s *t* test. A value of *p* < 0.05 was considered statistically significant.

## Results

### DR30318 is a tri-specific fusion protein engineered to specifically target CLDN18.2, human serum albumin and CD3

**Fig. 1:**
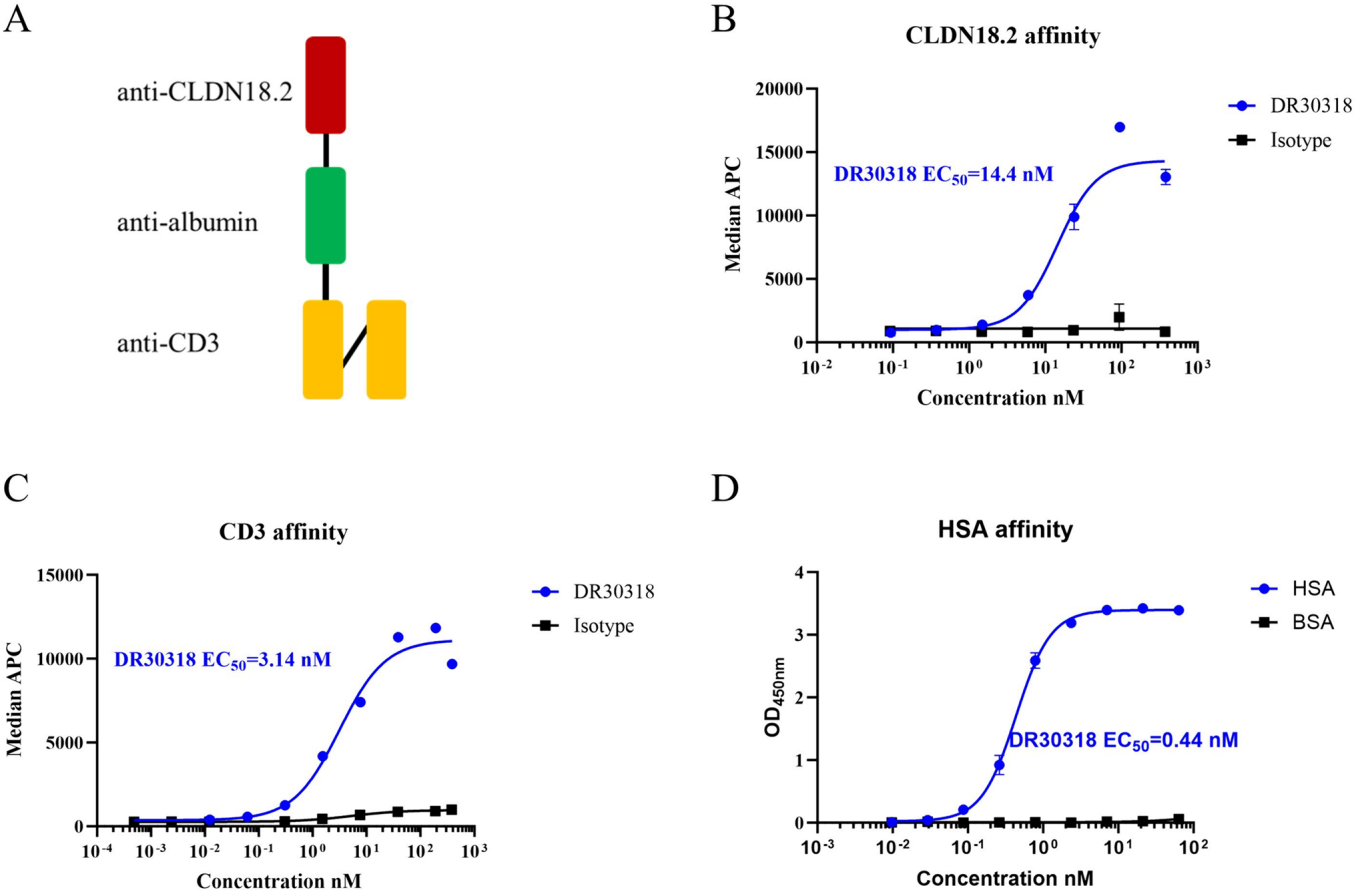
The structure and binding affinities of DR30318 in vitro. **A** Schematic diagram of DR30318 structure. DR30318 consists of an anti-CLDN18.2 domain (red), an anti-CD3 scFv domain (orange), an HSA binding domain (green) and two G_4_S linkers (black). **B** Binding affinity of DR30318 to human CLDN18.2. Serial dilutions of DR30318 (blue) and isotype control (black) were incubated with CHO-CLDN18.2-gfpx cells (over-expressing CLDN18.2) and detected by flow cytometry. The isotype control, structurally similar to DR30318, binds to HSA and CD3 but not to CLDN18.2. **C** Binding affinity of DR30318 to CD3. Serial dilutions of DR30318 (blue) and isotype control (black) were incubated with Jurkat (endogenously expressing CD3) and detected by flow cytometry. **D** Binding of DR30318 to HSA. Serial dilutions of DR30318 were incubated with plate-bound HSA (blue) or BSA (black) and detected using ELISA

Previously, we have isolated multiple anti-CLDN18.2 variable domains of heavy chain of heavy-chain antibodies (VHH) from an *alpaca* [35]. Utilizing the optimal VHH sequence, we successfully engineered DR30318 as a T cell engager with specific binding to CLDN18.2, HSA and CD3. As depicted in Fig. 1A, DR30318 is a 52.3 kDa molecule composed of an N-terminal anti-CLDN18.2 VHH domain, a C-terminal anti-CD3 scFv domain and an anti-HSA domain which is designed to enhance its in vivo half-life. The three binding domains monovalent bind to targets, respectively, and were connected via G_4_S linkers. The binding affinities of DR30318 to human CLDN18.2 and human CD3 were determined using flow cytometry, resulting in EC_50_ values of 14.4 nM and 3.14 nM (Fig. 1B, C), respectively. DR30318 exhibited strong affinity toward HSA with an EC_50_ value of 0.44 nM and no significant binding was observed with BSA (Fig. 1D).

### DR30318 exhibits potent TDCC activity in vitro

**Fig. 2:**
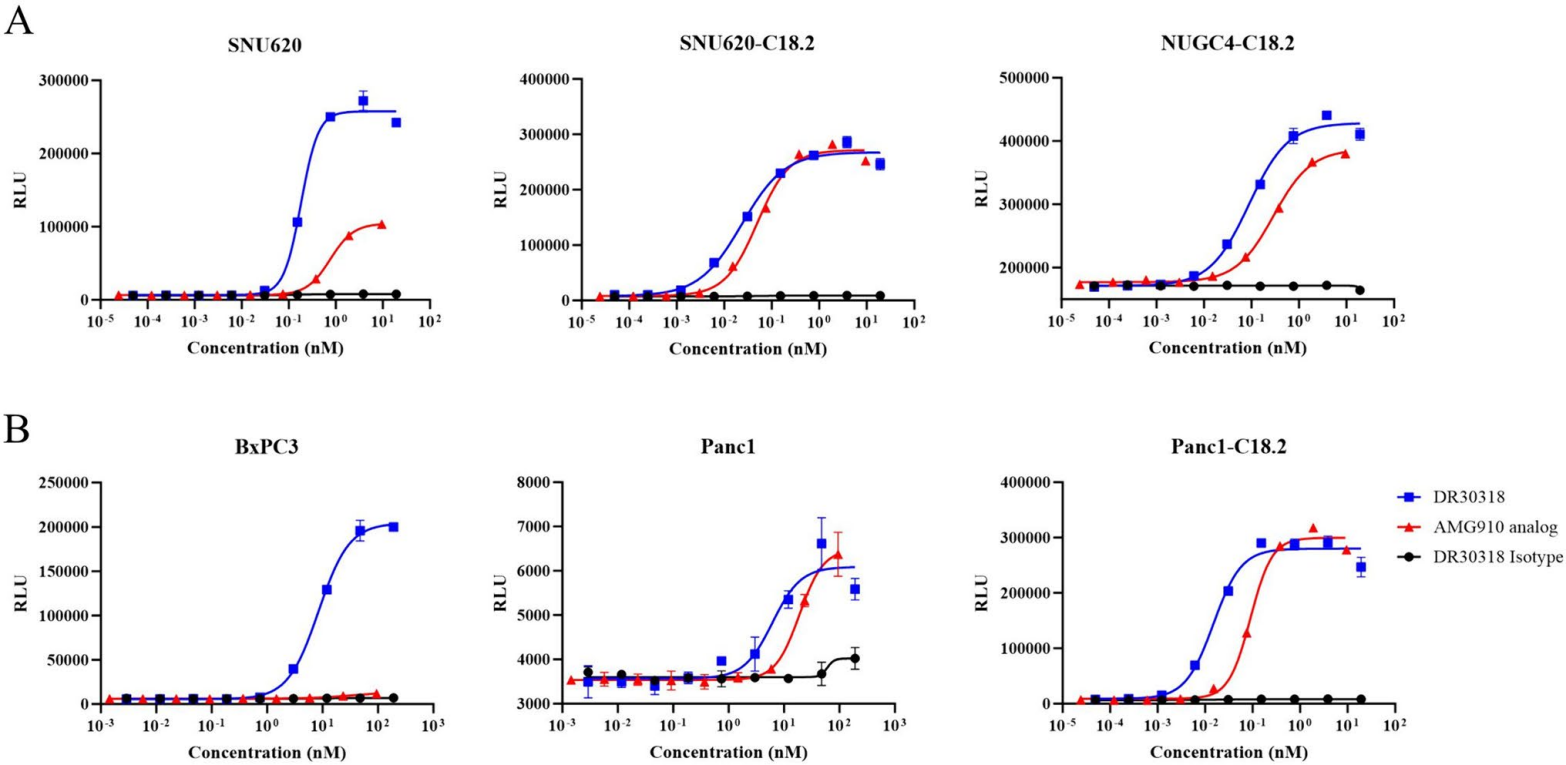
TDCC activities of DR30318 and AMG910 determined by the luciferase-based reporter gene assay. **A** Serial dilutions of DR30318 (blue), AMG910 (red) and isotype control (black) were incubated with CLDN18.2 expression gastric cancer cells (target cells) and Jurkat-NFAT-Luc (effector cells). **B** Serial dilutions of DR30318 (blue), AMG910 (red) and isotype control (black) were incubated with CLDN18.2 expression pancreatic cancer cells (target cells) and Jurkat-NFAT-Luc (effector cells)

Firstly, a luciferase reporter gene assay was employed to evaluate the TDCC activity of DR30318. Utilizing CHO-CLDN18.2-gfpx cells as target cells and Jurkat-NFAT-Luc cells as effectors, both DR30318 and AMG910 exhibited robust TDCC activities (Figure S1 in Supplementary materials II), with EC_50_ values of 0.047 nM and 0.062 nM, respectively. The isotype control, which binds to HSA and CD3 but not to CLDN18.2, exhibited no TDCC activity. Meanwhile, DR30318 did not show TDCC activity against CHO-K1Q and HEK293 cells which have no endogenous CLDN18.2 expression (Figure S2 in Supplementary materials II), thereby indicating a CLDN18.2 specific mechanism of action for DR30318. Gastric cancer cell lines SNU620, SNU620-C18.2, NUGC4-C18.2 and pancreatic cancer cell lines BxPC3, Panc1, Panc1-C18.2, which endogenously or stable exogenously transfection express CLDN18.2 (Figure S3 in Supplementary materials II), were utilized as the target cells. Against both gastric (Fig. 2A) and pancreatic cancer (Fig. 2B) cell lines, DR30318 was more potential especially against low or moderate CLDN18.2 expressing cells.

**Fig. 3:**
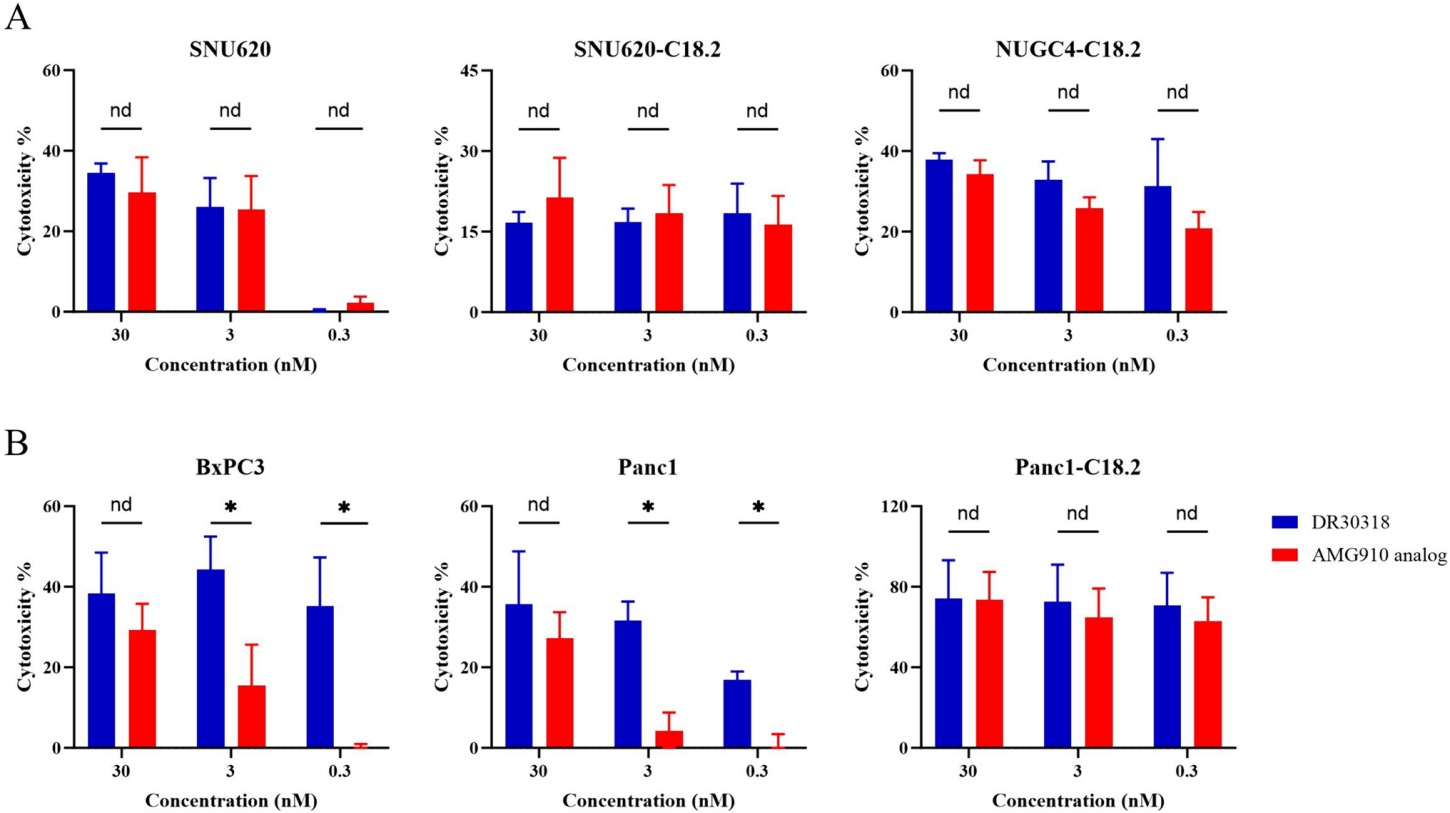
TDCC activities of DR30318 and AMG910 determined by the PBMC-based TDCC assay. **A** Serial dilutions of DR30318 (blue), AMG910 (red) and isotype control (black) were incubated with CLDN18.2 expression gastric cancer cells (target cells) and T cells isolated from PBMC (SNU620) or PBMCs (SNU620-C18.2 and NUGC4-C18.2) of two healthy donors (effector cells). **B** Serial dilutions of DR30318 (blue), AMG910 (red) and isotype control (black) were incubated with CLDN18.2 expression pancreatic cancer cells (target cells) and PBMCs of two healthy donors (effector cells). * indicated a significant difference of *p* < 0.05

Additionally, a PBMC-based TDCC assay was performed to evaluate the TDCC activity of DR30318. PBMCs obtained from two healthy volunteers were employed as the effector cells. Against both gastric (Fig. 3A) and pancreatic cancer (Fig. 3B) cell lines, DR30318 shows better TDCC potential especially against low or moderate CLDN18.2 expressing cells. After being incubated with equimolar protein, less cancer cells in DR30318 treatment group showed good stretched state (Figure S4 in Supplementary materials II). These data indicated the promising TDCC activity of DR30318 and the need for in vivo assessment.

### DR30318 enhances T cell proliferation in vitro

**Fig. 4:**
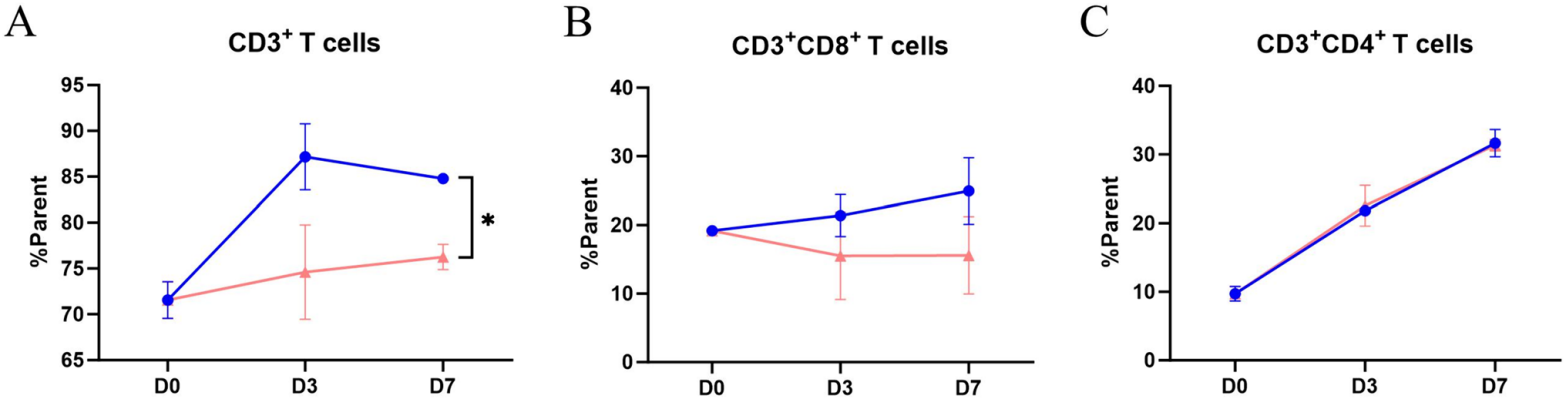
The T cell stimulation effect of DR30318 in vitro. PBMCs of 2 healthy donors were seeded into 96-well U type plate and being incubated with DR30318 (blue) and AMG910 (red) for 0, 3 and 7 days. CD3^+^ T cells (**A**), CD3^+^ CD8^+^ T cells (**B**) and CD3^+^ CD4^+^ T cells (**C**) were stained and analyzed with NovoCyto 2060R (Aligent). * indicated a significant difference of *p* < 0.05 at day 7

After being incubated for 3 or 7 days, the proportion of CD3^+^ T cells slightly increased when treated with DR30318 compared to those treated with AMG910. However, there was no significant difference observed in the levels of CD4^+^ and CD8^+^ T cells between incubation with DR30318 and AMG910 (Fig. 4).

### DR30318 pharmacokinetic profiles in rodents and non-human primate

**Fig. 5:**
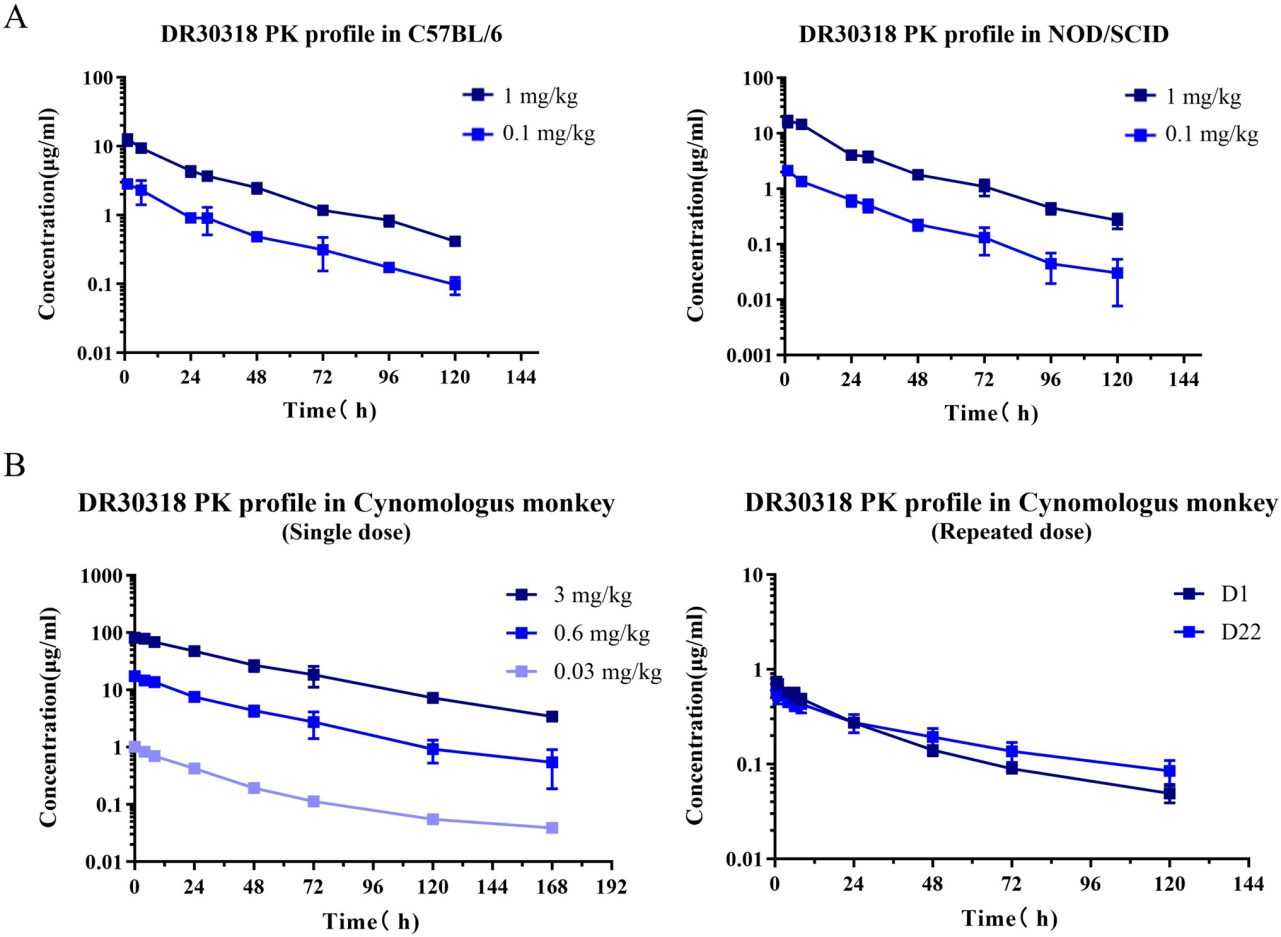
The pharmacokinetic profiles of DR30318 in vitro. **A** The pharmacokinetic profiles of DR30318 in C57BL/6 mice. After the single dose administration of DR30318 at 0.1 mg/kg (blue) or 1 mg/kg (dark blue), the serum concentrations were determined at specified time points using ELISA. **B** The pharmacokinetic profiles of DR30318 in NOD/SCID mice. After the single dose administration of DR30318 at 0.1 mg/kg (blue) or 1 mg/kg (dark blue), the serum concentrations were determined at specified time points using ELISA. **C** The pharmacokinetic profiles of DR30318 after a single dose administration in cynomolgus monkeys. After the administration of a single dose of DR30318 at 0.03 mg/kg (light blue), 0.6 mg/kg (blue) or 3 mg/kg (dark blue), serum concentrations were determined at specified time points using ELISA. **D**. The pharmacokinetic profiles of DR30318 after a repeat dose administration in cynomolgus monkeys. After four cycles of repeated administration of DR30318 at 0.03 mg/kg, serum concentrations were determined at specified time intervals using ELISA

The pharmacokinetic profiles of DR30318 were first evaluated in vivo in both rodents (Fig. 5A) and non-human primates (Fig. 5B). Following single intravenous administration of doses at 0.1 and 1 mg/kg, DR30318 exhibited nonlinear pharmacokinetics in C57BL/6 mice with a half-life of 28.6 ± 0.3 h, and the drug exposure ratio was lower than the dose ratio (Table 1). However, in NOD/SCID mice which lacks immune cell, DR30318 demonstrated nearly linear pharmacokinetics with a half-life of 22.2 ± 2.3 h. These results provided the basis for dosage design in pharmacodynamics studies.

**Table 1:** Pharmacokinetic parameters of DR30318 in rodents

Parameter^*^	Unit	C57BL/6		NOD-SCID	
		0.1 mg/kg (*n* = 8)	1 mg/kg (*n* = 8)	0.1 mg/kg (*n* = 8)	1 mg/kg (*n* = 8)
*t*_1/2_	h	28.40	28.81	20.53	23.85
*C*_max_	μg/ml	2.83	12.70	2.12	17.06
AUC_0-t_	μg/ml*h	81.15	355.30	45.96	399.19
AUC_0-∞_	μg/ml*h	85.13	372.60	46.86	408.46
Vz	mL/kg	48.13	111.55	63.22	84.23
CL	mL/(h·kg)	1.17	2.68	2.13	2.45
MRT_0-∞_	h	34.85	35.30	25.55	24.85
Dose ratio		1 : 10		1 : 10	
*C*_max_ ratio		1 : 4.5		1 : 8.1	
AUC_0-t_ ratio		1 : 4.4		1 : 8.7	
AUC_0-∞_ ratio		1 : 4.4		1 : 8.7	

*The PK parameters were derived from the mean concentration at each time point

After intravenous administration of 0.03, 0.6 and 3 mg/kg, DR30318 exhibited linear pharmacokinetics in cynomolgus monkeys (Fig. 5B and Table 2), with a half-life of 41.8 ± 8.7 h. No significant sex-related differences were observed, as evidenced by the exposure ratio of *C*_max_ and AUC being close to 1.0. The accumulation of DR30318 was not evident after 4-week doses of DR3018 (Table 1 in Supplementary materials I).

**Table 2:** Pharmacokinetic parameters of DR30318 in Cynomolgus macaques

Parameter	Unit	0.03 mg/kg		0.6 mg/kg		3 mg/kg	
		Male (*n* = 1)	Female (*n* = 1)	Male (*n* = 1)	Female (*n* = 1)	Male (*n* = 1)	Female (*n* = 1)
*t*_1/2_	h	34.54	57.65	36.62	38.15	45.87	37.73
*C*_max_	μg/ml	1.04	1.00	16.87	17.78	82.73	79.28
AUC_0-t_	μg/ml*h	33.03	33.16	569.18	714.36	3494.02	4198.06
AUC_0-∞_	μg/ml*h	35.17	36.07	584.66	758.33	3699.57	4404.15
Vz	mL/kg	42.50	69.18	54.22	43.55	53.66	37.08
CL	mL/(h·kg)	0.85	0.83	1.03	0.79	0.81	0.68
MRT_0-∞_	h	48.10	56.26	37.84	53.24	50.65	53.16
Dose ratio		1 : 20.0 : 100.0					
*C*_max_ ratio		1 : 17.0 : 79.4					
AUC_0-t_ ratio		1 : 19.4 : 116.0					
AUC_0-∞_ Ratio		1 : 18.9 : 113.8					

### Cytokines release assay of DR30318

Cytokine release syndrome (CRS) frequently occurs after immunotherapy treatment that activates T cells, particularly when employing the T cell engager drug format. Therefore, in the PK study and an in vitro PBMC stimulation assay, we assessed the cytokine release induced by DR30318 administration.

**Fig. 6:**
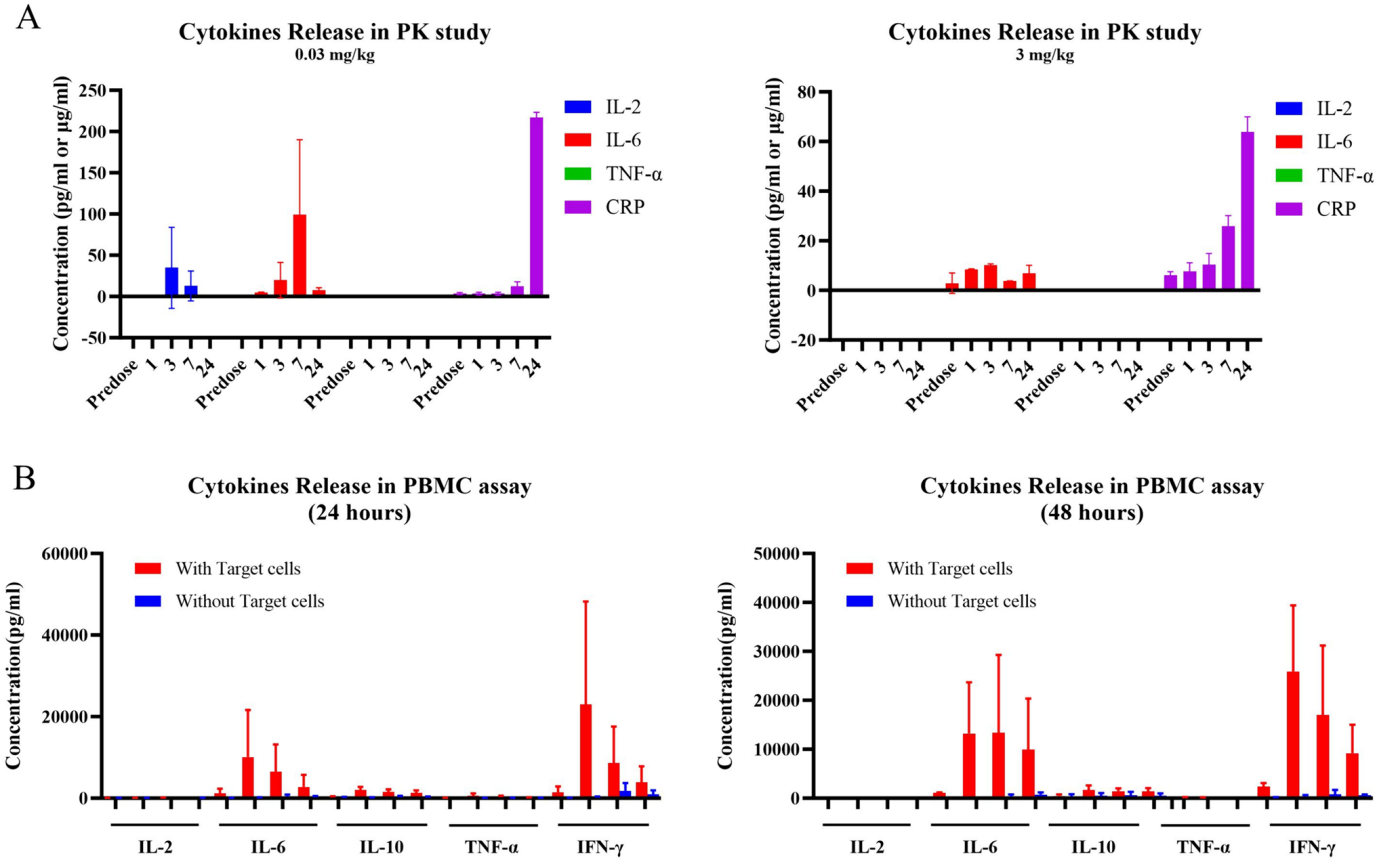
Cytokines release induced by DR30318. **A** Cytokines in PK samples of 0.03 mg/kg group. PK samples were collected at predose, 1 h, 3 h, 7 h and 24 h time points and analyzed for the levels of IL-2 (blue histogram), IL-6 (red histogram), TNF-α (green histogram) and CRP (violet histogram). Data are displayed with Mean ± SD (*n* = 3). The concentration of IL-2, IL-6 and TNF-α was demonstrated in pg/ml and CRP in μg/ml. **B** Cytokines in PK samples of 3.0 mg/kg group. PK samples were collected at predose, 1 h, 3 h, 7 h and 24 h time points and analyzed for the levels of IL-2 (blue histogram), IL-6 (red histogram), TNF-α (green histogram) and CRP (violet histogram). Data are displayed with Mean ± SD (*n* = 3). The concentration of IL-2, IL-6 and TNF-α was demonstrated in pg/ml and CRP in μg/ml. **C** DR30318 was incubated with PBMC effector cells (*n* = 2) and CHO-CLDN18.2-gfpx target cells at concentrations of 0, 0.025, 0.5 and 10 μg/ml for 24 h. The red histogram represents the presence of target cells, while the blue histogram represents their absence. The culture supernatant was collected, and the IL-2, IL-6, IL-10, TNF-α and IFN-γ levels were quantified. **D** DR30318 was incubated with PBMC effector cells (*n* = 2) and CHO-CLDN18.2-gfpx target cells at 0, 0.025, 0.5 and 10 μg/ml concentrations for 48 h. The red histogram represents the presence of target cells, while the blue histogram represents their absence. The culture supernatant was collected, and the IL-2, IL-6, IL-10, TNF-α and IFN-γ levels were quantified

The serum samples from the PK study of 0.03 mg/kg and 3 mg/kg groups were analyzed for the production of cytokines. As depicted in Fig. 6A and Table 2 in Supplementary materials I, DR30318 elicited a transient elevation of IL-6 levels at the third hour in the 0.03 mg/kg group or at the seventh hour in the 3 mg/kg group, which subsequently returned to baseline by 24 h. The administration of DR30318 at a dosage of 0.03 mg/kg resulted in a slight increase in IL-2 levels between the third- and seventh-hour post-administration. However, continuous elevation of CRP was observed following DR30318 administration, while no production of TNF-α was detected.

Furthermore, DR30318 was incubated with human PBMCs from two healthy donors in the presence or absence of target cells to assess the potential risk of CRS. As depicted in Fig. 6B and Table 3 in Supplementary materials I, DR30318 elicited robust production of IL-6 and IFN-γ as well as trace amounts of IL-2, IL-10 and TNF-α when CHO-CLDN18.2-gfpx target cells were present. Conversely, without target cells, DR30318 did not induce significant cytokine production.

### DR30318 treatment induces tumor regression in xenograft models

PBMC-based humanized xenograft models were utilized to investigate the tumor growth inhibitory effects of DR30318. The CLDN18.2 expression of the xenograft models was measured using immunohistochemical staining method, also was the CD3 positive cell in the tumor tissues. In our previous study, the CD3 positive rate in anti-CD3 scFv fusion protein treatment group showed no significant increase compared with vehicle group (data not shown), though the T cell stimulation assay using PBMCs from healthy donors showed that DR30318 could promote CD3^+^ T cell proliferation (Fig. 4A), so we monitored the tumor-infiltrating CD3 T cells at the final of study instead of dynamic comparison.

**Fig. 7:**
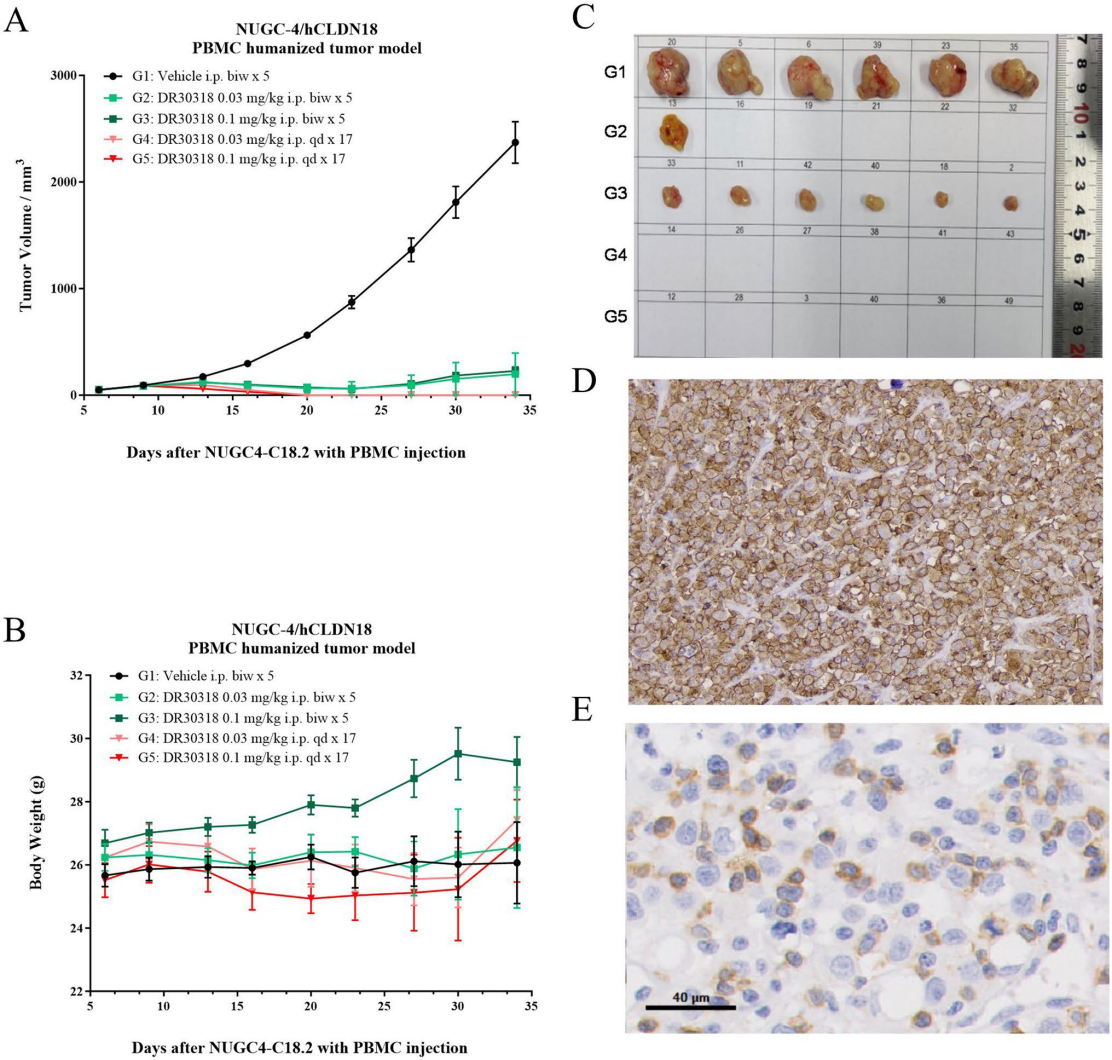
Tumor growth inhibition of DR30318 in human gastric cancer xenograft model. **A** Tumor growth curves of NUGC4/hCLDN18.2 bearing mice injected with different formulations. **B** Body weight changes of NUGC4/hCLDN18.2 bearing mice injected with different formulations. Data are shown as mean ± SEM, *n* = 6, ***p < 0.001 (vs. Vehicle). **C** Representative tumor images treated with different formulations were captured at the study endpoint. **D** Representative image of CLDN18.2 expression in tumor of vehicle group at the study endpoint. **E** Representative image of CD3 expression in tumor of vehicle group at the study endpoint

In the gastric cancer NUGC4/hCLDN18.2 PBMC humanized tumor model, DR30318 demonstrated potent tumor inhibitory efficacy (Fig. 7A). In the QD dosing group, complete regression of tumors was observed with DR30318 at the end of the study. Furthermore, in the BIW dosing group, administration of 0.03 mg/kg and 0.1 mg/kg of DR30318 exhibited potent tumor inhibition efficacy, resulting in complete tumor remission in five out of six mice and significant tumor regression in all six mice (Fig. 7C). The body weight did not show any significant differences among the five groups (Fig. 7B), indicating that DR30318 holds promise as a safe and effective therapeutic candidate for gastric cancer treatment.

**Fig. 8:**
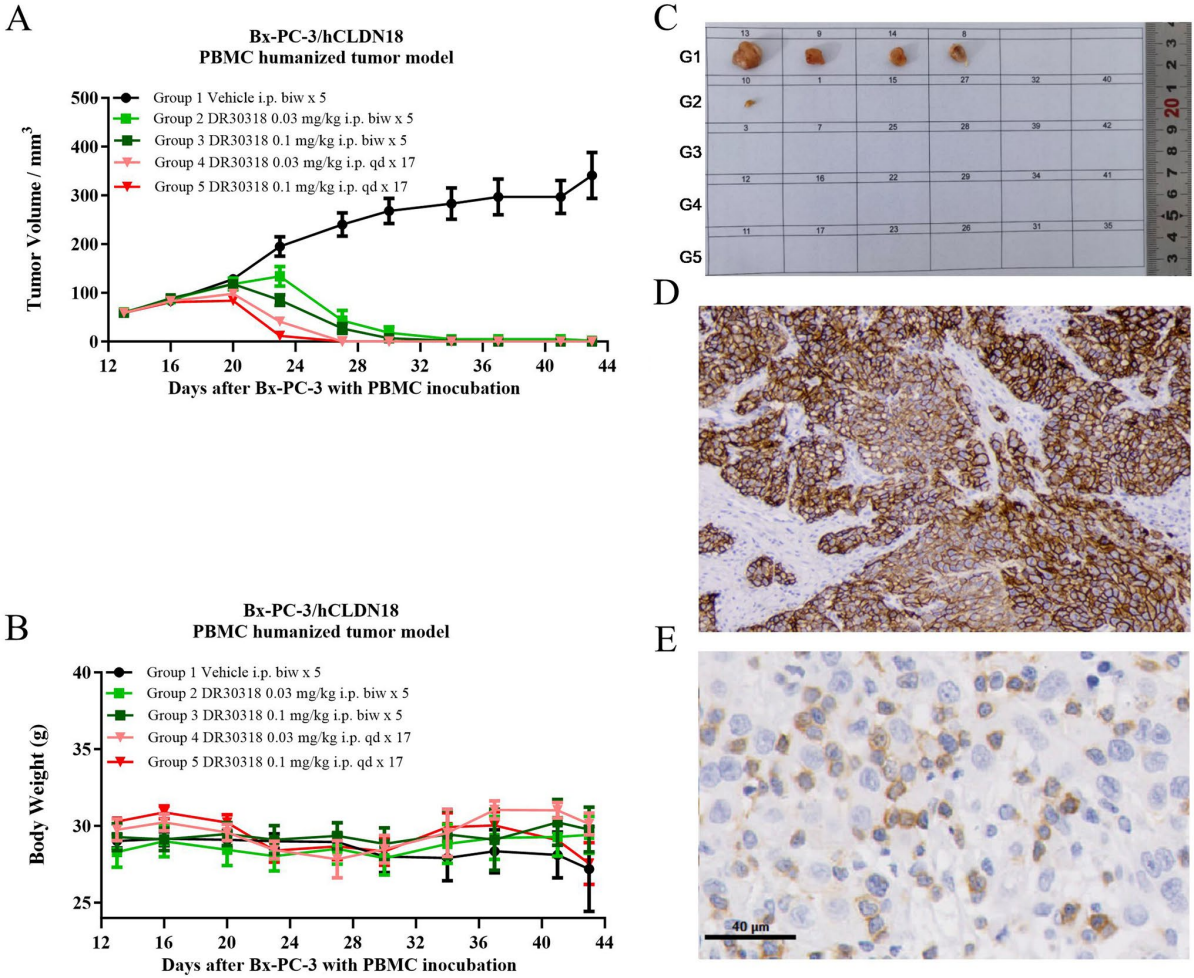
Tumor growth inhibition of DR30318 in human pancreatic cancer xenograft model. **A** Tumor growth curves of BxPC3/hCLDN18.2 bearing mice injected with different formulations. **B** Body weight changes of BxPC3/hCLDN18.2 bearing mice injected with different formulations. Data are shown as mean ± SEM, *n* = 6, ***p < 0.001 (vs. Vehicle). **C** Representative tumor images treated with different formulations were captured at the study endpoint. **D** Representative image of CLDN18.2 expression in tumor of vehicle group at the study endpoint. **E** Representative image of CD3 expression in tumor of vehicle group at the study endpoint

In the pancreatic cancer BxPC3/hCLDN18.2 PBMC humanized tumor model, DR30318 demonstrated a significant antitumor effect (Fig. 8A). From day 20 to day 34, there was an observed dose-dependent and frequency-dependent inhibitory effect on tumor growth. By the end of the study on Day 34, DR30318 nearly achieved complete regression of tumors in all treatment groups (Fig. 8C). Meanwhile, the administration of DR30318 did not elicit any detrimental impact on body weight across all experimental groups (Fig. 8B), thereby establishing DR30318 as a safe and productive therapeutic candidate for treating pancreatic cancer.

## Discussion

Globally, gastric and pancreatic cancers rank among the most prevalent malignancies and are leading contributors to mortality in oncology [39, 40]. Due to the scarcity of effective early diagnosis, a considerable number of individuals are diagnosed with advanced or metastatic disease, resulting in a 5-year survival rate below 20% for gastric cancer and merely 2% for pancreatic cancer [41, 42]. With the induction of combination chemotherapy such as gemcitabine and nab-paclitaxel or FOLFIRINOX, the 5-year survival reached up to approximately 11% in 2021 [43], pancreatic cancer is still the most dismal disease. Consequently, substantial unmet clinical needs for both gastric and pancreatic cancers exist. CLDN18.2 demonstrates aberrant overexpression in various malignancies, including gastric and pancreatic cancer; however, its expression is strictly confined to gastric mucosal cells in healthy tissues, rendering it a promising therapeutic target for CLDN18.2 positive cancers.

The investigational Zolbetuximab, a first-in-class monoclonal antibody specifically targeting CLDN18.2, demonstrated promising results in Astellas's Phase III SPOTLIGHT trial. Combination therapy with Zolbetuximab and mFOLFOX6 resulted in a significant improvement in median progression-free survival (8.21 months VS 6.80 months) and a notable reduction in the risk of mortality (hazard ratio [HR] 0.75, 95% confidence interval [CI] 0.60–0.94) [44]. However, Zolbetuximab demonstrates efficacy exclusively in patients exhibiting robust CLDN18.2 expression (≥ 75% of tumor cells displaying moderate-to-strong membranous staining for CLDN18.2). Therefore, there remains an urgent need to explore novel therapeutic modalities targeting CLDN18.2 positive cancers.

T cell engagers (TCEs) hold great promise as a therapeutic modality, enabling the redirection of T cells toward specific tumor antigens. AMG910, developed on AMGEN's HLE platform, represents the pioneering clinical entry of a CLDN18.2-targeting TCE [45]. In our study, we successfully developed a tri-specific TCE, DR30318, which exhibited simultaneous binding to CLDN18.2, HSA and CD3 in vitro affinity assays. This unique characteristic of DR30318 induced potent TDCC activity in vitro as demonstrated by both reporter gene TDCC assay and PBMC-based TDCC assay using T cells isolated from PBMC or PBMCs of healthy donors. Its potent TDCC effect against low or moderate CLDN18.2 expressing cells even in lower concentration made it exciting for the antigen degradation during course of disease or therapy. Moreover, the HSA binding domain (with serum albumin affinity from high to low: human ≈ cynomolgus monkey > mouse, data not shown) endowed DR30318 with a relatively longer half-life of 22.2–28.6 h in rodents and 41.8 h in cynomolgus monkey compared to canonical BiTE. Unlike most Fc-based TCEs, DR30318 is a monomeric fusion protein that possesses several appealing characteristics, including facile manufacturability and enhanced penetration into solid tumor tissues due to its low molecular weight.

Cytokine release syndrome (CRS) is a commonly observed adverse event in T cell engaging immunotherapies, particularly for TCEs, with IL-6 playing a pivotal role in the pathophysiology of CRS [46]. In the in vivo PK study conducted on cynomolgus monkeys, administration of DR30318 at doses of 0.03 mg/kg and 3 mg/kg resulted in a transient and slight increase in IL-6 production, which remained significantly lower than the level reported in certain publication [47]. The C-reactive protein (CRP) demonstrates a rapid response to IL-6 stimulation and can serve as a reliable biomarker for monitoring the progression of CRS [48]. In our study, administration of DR30318 resulted in a sustained elevation of CRP levels, potentially indicating an on-target off-tumor toxicity in the stomach, as evidenced by observed gastric-related adverse effects such as anorexia, nausea and mucosal damage (data not shown). However, DR30318 did not induce significant production of TNF-α and IL-2. In vitro coculture with human PBMCs demonstrated that DR30318 elicited potent TDCC activities and triggered the release of IL-6 and IFN-γ in the presence of target cells. Conversely, in the absence of target cells, DR30318 failed to induce cytokine production, thereby suggesting a mechanism of action dependent on binding to CLDN18.2 and minimizing the risk of peripheral immune system activation.

DR30318 demonstrated potent tumor growth inhibition in both gastric and pancreatic xenograft models. A daily dosage of 0.03 mg/kg in the gastric cancer model effectively suppressed tumor growth. In the pancreatic cancer model, treatment with DR30318 nearly resulted in complete tumor regression by the end of the study period. Significantly, administration of DR30318 did not adversely affect body weight in mice, indicating its safety profile and potential as a promising therapeutic candidate for gastric and pancreatic cancers. Considering that DR30318 induced only mild IL-6 production at 0.03 mg/kg and 3 mg/kg, it suggests a wide therapeutic window for DR30318, which is a crucial characteristic for most therapeutic compounds targeting TCEs.

In summary, we successfully constructed a tri-specific T cell engager, DR30318. In vitro and in vivo studies demonstrated CLDN18.2 target dependent TDCC activities to kill the tumor cells. The lower degree of cytokine release and robust tumor regression activity indicated that DR30318 was a promising therapeutic agent for CLDN18.2 positive cancers.

### Supplementary Information

Below is the link to the electronic supplementary material.

Supplementary file1 (DOCX 34 kb)

Supplementary file2 (DOCX 612 kb)
