# Supplementary material for: DR30318, a novel tri-specific T cell engager for Claudin 18.2 positive cancers immunotherapy
Source: Cancer Immunol Immunother. 2024 Mar 30;73(5):82. doi: 10.1007/s00262-024-03673-x (PMC10981630; doi:10.1007/s00262-024-03673-x)
Supplement: Supplementary file 1 — Supplementary file1 (DOCX 34 kb) [file 262_2024_3673_MOESM1_ESM.docx]

**Supplementary materials Ⅰ**

**Table 1**. Pharmacokinetic parameters of repeated dose of DR30318 in Cynomolgus monkeys.

| Parameter | Unit | D1 | | | | D22 | | | |
| --- | --- | --- | --- | --- | --- | --- | --- | --- | --- |
|  |  | Male(n=2) | | Female(n=2) | | Male(n=2) | | Female(n=2) | |
| t_1/2_ | h | 27.29 | 58.03 | 33.75 | 62.95 | 42.9 | 83.5 | 54.2 | 62.7 |
| T_max_ | h | 0.5 | 0.5 | 0.5 | 0.5 | 0.5 | 0.5 | 0.5 | 0.5 |
| C_max_ | µg/mL | 0.66 | 0.9 | 0.69 | 0.82 | 0.57 | 0.71 | 0.47 | 0.66 |
| AUC_0-last_ | h·µg/mL | 18.41 | 28.78 | 20.99 | 18.87 | 21.89 | 33.9 | 16.9 | 24.03 |
| AUC_0-∞_ | h·µg/mL | 19.65 | 35.45 | 22.52 | 23.55 | 25.84 | 50.71 | 20.7 | 32.02 |
| Vz | mL/kg | 60.1 | 70.85 | 64.87 | 115.68 | 71.89 | 71.28 | 113.31 | 84.82 |
| Cl | mL/(h·kg) | 1.53 | 0.85 | 1.33 | 1.27 | 1.16 | 0.59 | 1.45 | 0.94 |
| MRT_0-∞_ | h | 37.17 | 66.94 | 39.65 | 69.27 | 61.27 | 108.27 | 67 | 83.77 |

**Table 2**. Cytokine release after single dose of DR30318 in Cynomolgus monkey at specified time points.

| Cytokines | Sampling Time(h) | **Dosage（mg/kg）** | | | |
| --- | --- | --- | --- | --- | --- |
|  |  | 0.03 | | 6 | |
| IL-2 (pg/ml) | predose | BLQ | BLQ | BLQ | BLQ |
|  | 1 | BLQ | BLQ | BLQ | BLQ |
|  | 3 | BLQ | 69.54 | BLQ | BLQ |
|  | 7 | BLQ | 25.52 | BLQ | BLQ |
|  | 24 | BLQ | BLQ | BLQ | BLQ |
| IL-6 (pg/ml) | predose | BLQ | BLQ | BLQ | 5.82 |
|  | 1 | 5.23 | 4.38 | 8.29 | 8.61 |
|  | 3 | 4.53 | 35.11 | 9.86 | 10.58 |
|  | 7 | 35.11 | 163.59 | 3.73 | 3.87 |
|  | 24 | 5.99 | 9.86 | 4.53 | 9.16 |
| TNFα (pg/ml) | predose | BLQ | BLQ | BLQ | BLQ |
|  | 1 | BLQ | BLQ | BLQ | BLQ |
|  | 3 | BLQ | BLQ | BLQ | BLQ |
|  | 7 | BLQ | BLQ | BLQ | BLQ |
|  | 24 | BLQ | BLQ | BLQ | BLQ |
| CRP (μg/ml) | predose | 2.52 | 4.40 | 5.34 | 7.14 |
|  | 1 | 2.19 | 4.72 | 5.34 | 10.13 |
|  | 3 | 1.90 | 4.62 | 7.16 | 13.56 |
|  | 7 | 9.15 | 16.31 | 22.87 | 28.86 |
|  | 24 | 212.69 | 221.44 | 59.64 | 68.19 |

BLQ, Below lower limit of quantification

**Table 3**. Cytokine release levels induced by DR30318 after incubation with PBMCs, with the absence or presence of CLDN18.2 positive cells.

| Cytokines | DR30318 Concentration | **Donor XW0809005** | | | | | | |  |
| --- | --- | --- | --- | --- | --- | --- | --- | --- | --- |
| (pg/ml) | (μg/ml) | 24h T+ | | 24h T- | | 48h T+ | | 48h T- | |
| IL-2 | 0 | 61.76 | 78.76 | 42.10 | 60.98 | 9.04 | 9.42 | 13.56 | 13.38 |
|  | 0.025 | 80.72 | 88.43 | 32.60 | 42.67 | 21.10 | 8.82 | 6.08 | BLQ |
|  | 0.5 | 201.00 | 148.56 | 28.71 | 44.10 | 19.96 | 14.00 | 11.14 | 10.86 |
|  | 10 | 125.98 | 132.11 | 93.42 | 93.17 | 22.74 | 24.66 | 18.96 | 50.96 |
| IL-6 | 0 | 1380.63 | 2571.6 | 27.9 | 18.57 | 1202.6 | 1137 | BLQ | BLQ |
|  | 0.025 | 20646.72 | 15810 | 401.52 | 33.54 | 24156.2 | 17061.8 | BLQ | BLQ |
|  | 0.5 | 9889.32 | 12589.17 | 113.25 | 179.46 | 25071.2 | 24138.6 | BLQ | 52.8 |
|  | 10 | 4049.37 | 5636.79 | 380.01 | 652.89 | 15767.6 | 18813.8 | 534.2 | 1577.6 |
| IL-10 | 0 | 278.7 | 188.34 | 3.63 | 2.01 | 273.2 | 211.2 | 82.4 | 12.6 |
|  | 0.025 | 1672.56 | 1363.29 | 141.9 | 8.91 | 1053.2 | 862.4 | 32.2 | 67 |
|  | 0.5 | 1341.6 | 964.95 | 74.82 | 103.71 | 980 | 1080.6 | 25.2 | 41.6 |
|  | 10 | 919.5 | 932.25 | 238.23 | 257.79 | 1011.8 | 871.6 | 186.8 | 185.8 |
| TNFα | 0 | 133.83 | 95.4 | BLQ | BLQ | BLQ | BLQ | BLQ | BLQ |
|  | 0.025 | 1140.21 | 837.96 | 58.23 | BLQ | 262 | 161.6 | BLQ | BLQ |
|  | 0.5 | 476.28 | 580.14 | 9.54 | 179.79 | 166.6 | 144 | BLQ | BLQ |
|  | 10 | 184.02 | 232.14 | 62.94 | 151.35 | 107.2 | 171.6 | 50.6 | 81.2 |
| IFN-γ | 0 | 2541.84 | 2370.36 | 27.42 | 89.52 | 2959.00 | 2847.40 | 321.80 | 121.40 |
|  | 0.025 | 43500.00 | 38155.59 | 618.72 | 162.06 | 44752.60 | 26146.40 | 72.80 | 89.80 |
|  | 0.5 | 15982.08 | 13922.31 | 689.91 | 5681.37 | 24926.20 | 29142.80 | 40.80 | 144.20 |
|  | 10 | 5073.87 | 8276.43 | 969.18 | 2310.48 | 14301.40 | 12301.00 | 728.40 | 765.80 |
|  |  |  |  |  |  |  |  |  |  |
| Cytokines | DR30318 Concentration | **Donor XW0809006** | | | | | | | |
| (pg/ml) | (μg/ml) | 24h T+ | | 24h T- | | 48h T+ | | 48h T- | |
| IL-2 | 0 | 33.89 | 25.52 | 81.47 | 56.74 | 6.38 | 12.98 | 23.56 | 17.06 |
|  | 0.025 | 45.14 | 59.86 | 81.93 | 61.13 | 10.64 | BLQ | 19.70 | 30.94 |
|  | 0.5 | 125.92 | 131.35 | 26.48 | 53.94 | 25.10 | 19.42 | 16.08 | 8.58 |
|  | 10 | 101.54 | 122.49 | 81.43 | 36.34 | 30.20 | 37.04 | 35.14 | 16.42 |
| IL-6 | 0 | 432.03 | 217.62 | 77.82 | 51.72 | 1252.6 | 829.8 | 155.8 | 87.4 |
|  | 0.025 | 1969.11 | 1823.4 | 233.64 | 101.82 | 7935.2 | 3744 | BLQ | 243 |
|  | 0.5 | 1397.49 | 2215.92 | 289.86 | 1192.74 | 1941.4 | 2280.4 | 513.8 | 774.4 |
|  | 10 | 768.72 | 567.39 | 554.52 | 194.85 | 2889 | 2145.4 | 611 | 257.2 |
| IL-10 | 0 | 432.69 | 397.29 | 316.92 | 182.49 | 768 | 578.8 | 850.8 | 543.8 |
|  | 0.025 | 2681.01 | 2400.03 | 167.01 | 260.82 | 2577.2 | 2061.6 | 775.4 | 988.6 |
|  | 0.5 | 2306.07 | 1721.67 | 565.59 | 382.44 | 1858 | 1862.6 | 1227.2 | 979.8 |
|  | 10 | 1824.27 | 1694.73 | 423.18 | 333.69 | 1810.8 | 1915.6 | 995.2 | 716.6 |
| TNFα | 0 | 35.49 | 32.61 | 29.04 | 21.36 | BLQ | BLQ | BLQ | BLQ |
|  | 0.025 | 114.3 | 107.55 | 30 | 13.56 | 70.8 | 78 | BLQ | BLQ |
|  | 0.5 | 53.1 | 82.68 | 94.41 | 38.37 | 53 | 51 | 39.6 | 43.2 |
|  | 10 | 25.35 | 23.16 | 110.82 | 56.22 | BLQ | BLQ | 46.6 | BLQ |
| IFN-γ | 0 | 406.86 | 349.89 | 116.94 | 68.91 | 1825.40 | 1856.00 | 220.20 | 151.20 |
|  | 0.025 | 5974.44 | 4261.89 | 137.40 | 161.79 | 21539.40 | 11142.80 | 961.00 | 156.80 |
|  | 0.5 | 2359.71 | 2198.61 | 473.25 | 523.14 | 7587.60 | 6350.20 | 616.40 | 2240.60 |
|  | 10 | 1260.21 | 1076.73 | 411.48 | 264.27 | 5823.40 | 4357.20 | 804.40 | 333.40 |

T+, with CHO-CLDN18.2-gfpx cells; T-, without CHO-CLDN18.2-gfpx cells; BLQ, below lower limit of quantification.
