# Supplementary material for: DR30318, a novel tri-specific T cell engager for Claudin 18.2 positive cancers immunotherapy
Source: Cancer Immunol Immunother. 2024 Mar 30;73(5):82. doi: 10.1007/s00262-024-03673-x (PMC10981630; doi:10.1007/s00262-024-03673-x)
Supplement: Supplementary file 2 — Supplementary file2 (DOCX 612 kb) [file 262_2024_3673_MOESM2_ESM.docx]

**Supplementary materials II**

**Figure S1. TDCC activities of DR30318 and AMG910 Analog determined by the luciferase-based reporter gene assay.** Serial dilutions of DR30318 (blue), AMG910 Analog (red) and isotype control (black) were incubated with CHO-C18.2-gfpx cells (target cells) and Jurkat-NFAT-Luc (effector cells).

**Figure S2. TDCC activities of DR30318 and AMG910 Analog determined by the luciferase-based reporter gene assay.** Serial dilutions of DR30318 (blue), AMG910 Analog (red) and isotype control (black) were incubated with HEK293 or CHO-K1Q cells (target cells) and Jurkat-NFAT-Luc (effector cells).

**
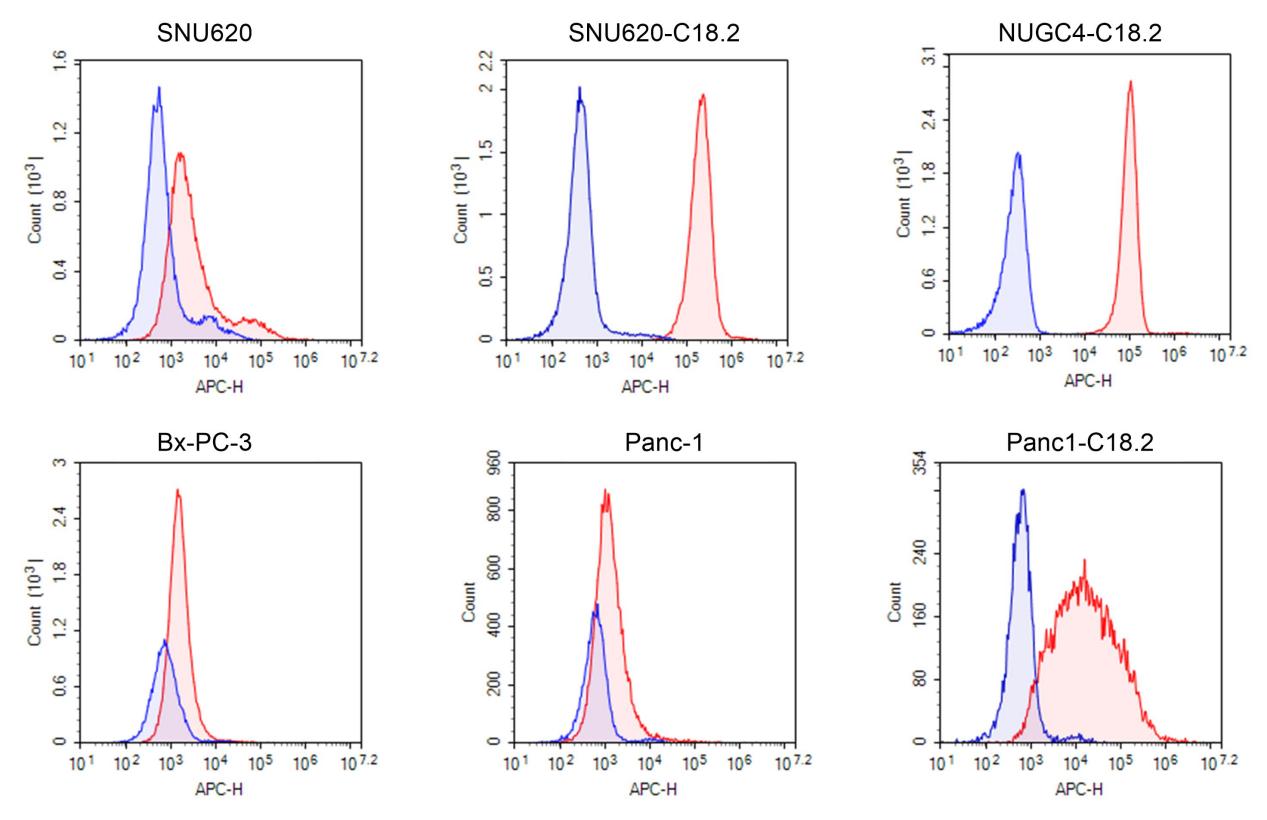
**

**Figure S3. CLDN18.2 expression of gastric or pancreatic cancer cell lines by flow cytometry.** Gastric or pancreatic cancer cell lines were harvested and seeded into 96 well U type microplate and incubate with DR30318 (red) or isotype control (blue) and analyzed with Novocyte 2060R(Agilent).


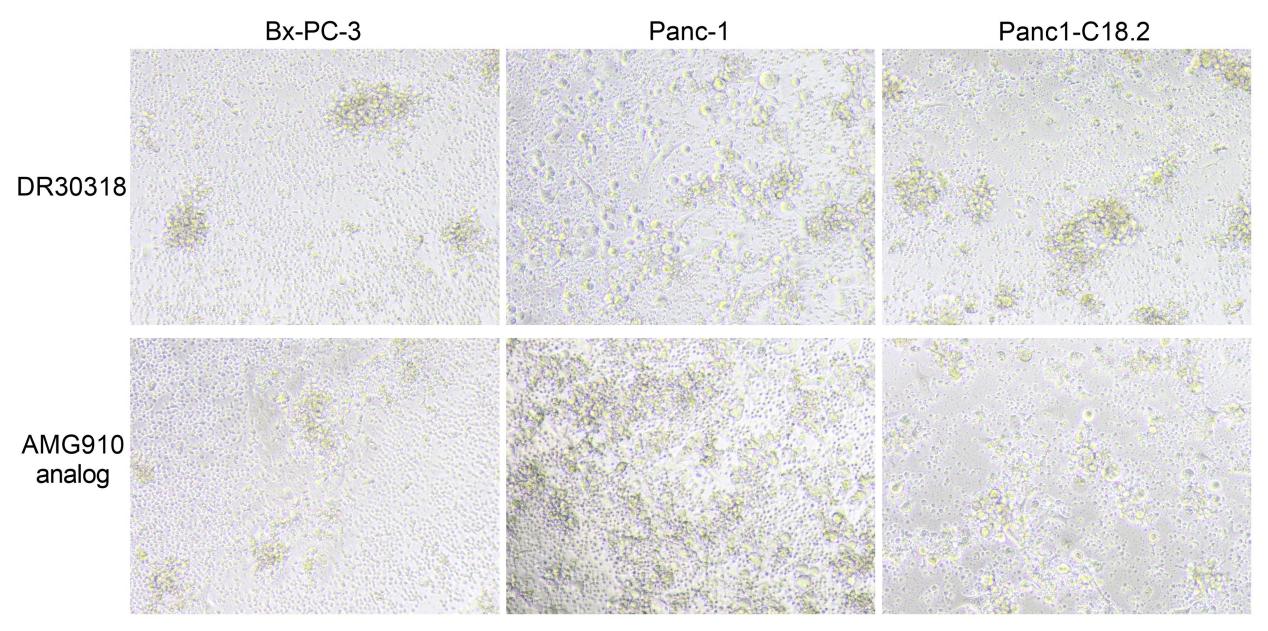


**Figure S4. TDCC activities of DR30318 and AMG910 Analog determined by the PBMC-based reporter gene assay.** 30 nM DR30318 and AMG910 Analog were incubated with Bx-PC-3, Panc-1 and Panc1-C18.2 cells (target cells) and PBMCs (effector cells). After incubate with equimolar protein, less cancer cells in DR30318 treatment group showed good stretched state. The small round cells were PBMCs while cancer cells were large in size.
